# Involvement of Bax and Bcl-2 in Induction of Apoptosis by Essential Oils of Three Lebanese *Salvia* Species in Human Prostate Cancer Cells

**DOI:** 10.3390/ijms19010292

**Published:** 2018-01-19

**Authors:** Alessandra Russo, Venera Cardile, Adriana C. E. Graziano, Rosanna Avola, Maurizio Bruno, Daniela Rigano

**Affiliations:** 1 Department of Drug Sciences, University of Catania, V.le A. Doria 6, 95125 Catania, Italy; 2 Department of Biomedical and Biotechnological Sciences, Section of Physiology, University of Catania, Via S. Sofia, 89, 95123 Catania, Italy; cardile@unict.it (V.C.); acegraz@unict.it (A.C.E.G.); rosanna.avola@unict.it (R.A.); 3 Department of Biological, Chemical and Pharmaceutical Sciences and Technologies (STEBICEF), University of Palermo, V.le delle Scienze, Parco d’Orleans II, 90128 Palermo, Italy; 4 Department of Pharmacy, University of Naples Federico II, Via D. Montesano, 49, 80131 Naples, Italy; drigano@unina.it

**Keywords:** *Salvia*, essential oil, prostate cancer, apoptosis, reactive oxygen species

## Abstract

Prostate cancer is one of the most common forms of cancer in men, and research to find more effective and less toxic drugs has become necessary. In the frame of our ongoing program on traditionally used *Salvia* species from the Mediterranean Area, here we report the biological activities of *Salvia aurea*, *S. judaica* and *S. viscosa* essential oils against human prostate cancer cells (DU-145). The cell viability was measured by 3(4,5-dimethyl-thiazol-2-yl)2,5-diphenyl-tetrazolium bromide (MTT) test and lactate dehydrogenase (LDH) release was used to quantify necrosis cell death. Genomic DNA, caspase-3 activity, expression of cleaved caspase-9, B-cell lymphoma 2 (Bcl-2) and Bcl-2 associated X (Bax) proteins were analyzed in order to study the apoptotic process. The role of reactive oxygen species in cell death was also investigated. We found that the three essential oils, containing caryophyllene oxide as a main constituent, are capable of reducing the growth of human prostate cancer cells, activating an apoptotic process and increasing reactive oxygen species generation. These results suggest it could be profitable to further investigate the effects of these essential oils for their possible use as anticancer agents in prostate cancer, alone or in combination with chemotherapy agents.

## 1. Introduction

Prostate cancer is one of the principal cause of death from cancer in older men and the most commonly diagnosed cancer in men overall [1]. Various treatments are available. Because prostate cancer is an androgen-sensitive tumor, androgen deprivation therapy is usually used for the treatment of early-stage prostate cancer. However, over time, prostate cancer growth becomes independent of androgen and renders androgen ablation therapy ineffective. At this stage the cancer is highly aggressive and metastasized [2,3]. Chemotherapy is used to treat castration-resistant prostate cancer, but it is not very effective and has many side effects [4]. Also for the novel strategies [5,6,7,8,9] that have shown promising results in castration-resistant prostate cancer patients, several adverse side effects have been observed [8,9]. Therefore, extensive research has been carried out in order to develop safer and more effective agents.

Medicinal plants have been used in healthcare since time immemorial, therefore in the last decades many natural products obtained from plants were tested for their anticancer efficacy. Several of these natural nontoxic compounds have been found to inhibit prostate cancer growth and metastasis, through different mechanisms. These results suggest that they can be considered a promising approach for the treatment of this cancer, and specifically for the advanced and androgen-independent stage of the malignancy [10,11,12].

Recently, the use of medicinal plants as alternative therapy for many cancers has been growing worldwide, and particularly in Lebanon and surrounding countries. The Lebanese people buy local medicinal plants in small stores called “Dabbous” in which the herbalists suggest plants for a specific disease without any form of prescription [13]. The genus *Salvia* is one of the most important aromatic and medicinal genera of the Lamiaceae family and comprises about 900 species [14], many of which are used in traditional medicine for the treatment of infections, malaria, inflammation and cancer [15]. Among the strongest active metabolites of sage there are the essential oils produced by the aerial parts (1–2.8%), whose main components are the monoterpenes α- and β-thujone, camphor, borneol and cineole, as well as the sesquiterpenes β-caryophyllene and α-humulene [16]. *Salvia aurea* L., *S. judaica* Boiss. and *S. viscosa* Jacq. are three *Salvia* species growing wild in Lebanon [17] and frequently found in multiherb products used in Middle East counties for the treatment of cancer and other diseases [18]. Recently, as part of our screening program of traditionally-used *Salvia* species from the Mediterranean Area [13,19,20,21,22,23], we have evidenced the ability of the essential oils from these three *Salvia* species to inhibit the growth of the human melanoma cells inducing apoptotic cell death [22]. On the basis of these promising results, in this paper we report the biological activity of *Salvia aurea* (Sa), *S. judaica* (Sj) and *S. viscosa* (Sv) essential oils against human androgen-insensitive prostate cancer cells DU-145. We found that the three essential oils are capable to reduce the growth of human prostate cancer cells, activating an apoptotic process and increasing reactive oxygen species generation.

## 2. Results

### 2.1. Cell Growth Inhibitory Effect of the Essential Oils

**Figure 1:**
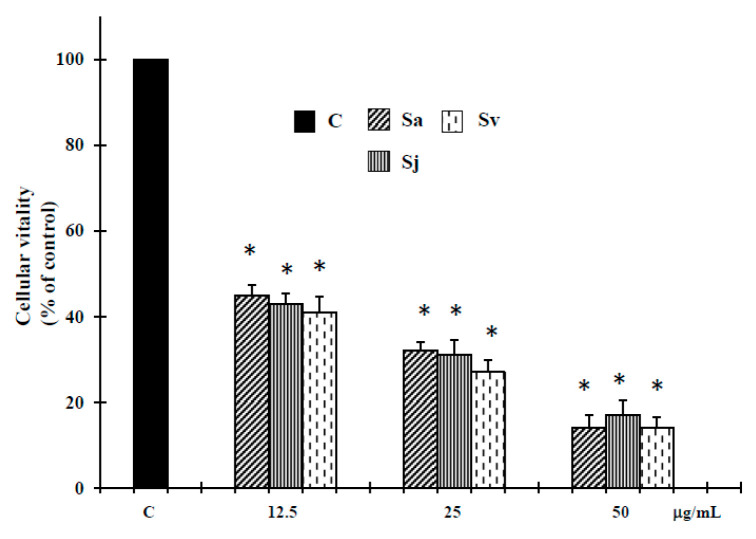
Cell growth, assayed using 3(4,5-dimethyl-thiazol-2-yl)2,5-diphenyl-tetrazolium bromide (MTT) test, of DU-145 cells untreated and treated with different concentrations of the essential oils from Sa, Sj and Sv for 72 h. The values are the mean ± standard deviation (SD) of three experiments performed in quadruplicate. * Significant vs. control untreated cells (*p* < 0.001).

The essential oils from aerial parts of Sa, Sj and Sv were tested in vitro for their potential human tumor cell growth inhibitory effect on DU-145 tumor cell line, using 3(4,5-dimethyl-thiazol-2-yl)2,5-diphenyl-tetrazolium bromide (MTT) assay. The results, summarized in Figure 1, show that all these natural products exhibited after 72 h of treatment a clear dose-response relationship in the range of 12.5–50 µg/mL concentrations. Interestingly, these concentrations, as published in our previous work [22], did not reveal cytotoxic effect against normal human buccal fibroblast cells, a cellular model used in toxicity studies [24,25].

### 2.2. Induction of Cell Death

No statistically significant increase in lactate dehydrogenase (LDH) release, used to quantify necrosis cell death [13], was observed in cancer cells treated with the essential oils at 12.5 and 25 µg/mL concentrations (Table 1).

Alternatively, we showed a significant LDH release at a higher concentration of 50 µg/mL (Table 1). Similar results were obtained with H_2_O_2_ (1000 µM), a necrotic inductor in cancer cell line, when it is used at high concentrations [26].

**Figure 2:**
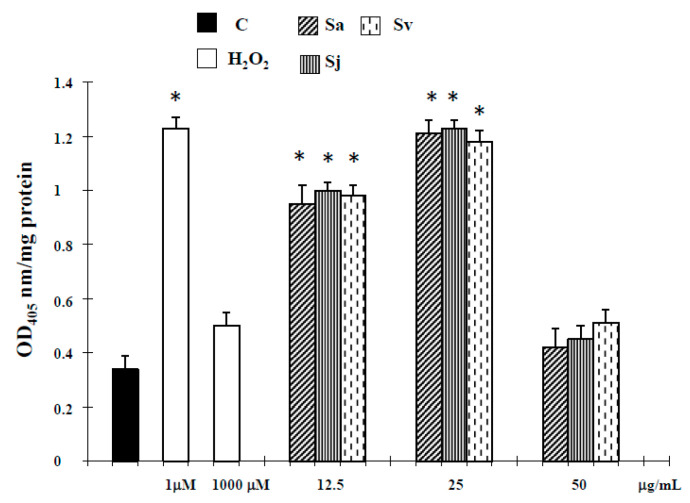
Caspase-3 activity, determined by using the Caspase colorimetric assay Kit (SIGMA RBI St. Louis, MO, USA), in DU-145 cells treated with different concentrations of the essential oils from Sa, Sj and Sv for 72 h. The values are the mean ± standard deviation (SD) of three experiments performed in quadruplicate. * Significant vs. control untreated cells (*p* < 0.001).

Dysregulation of apoptosis in cancer cells contributes to carcinogenesis and is involved in the resistance to cytotoxic anticancer drugs [27]. Therefore, to better discriminate between apoptosis and necrosis, the next experiments were performed to characterize the role of activation of caspase-3, the major executioner caspase in the caspase cascade [28]. As shown in Figure 2, the activity of caspase-3 was significantly increased in DU-145 cells treated for 72 h with the essential oils at concentration of 12.5 and 25 µg/mL, and hydrogen peroxide (H_2_O_2_) (1 µM).

**Figure 3:**
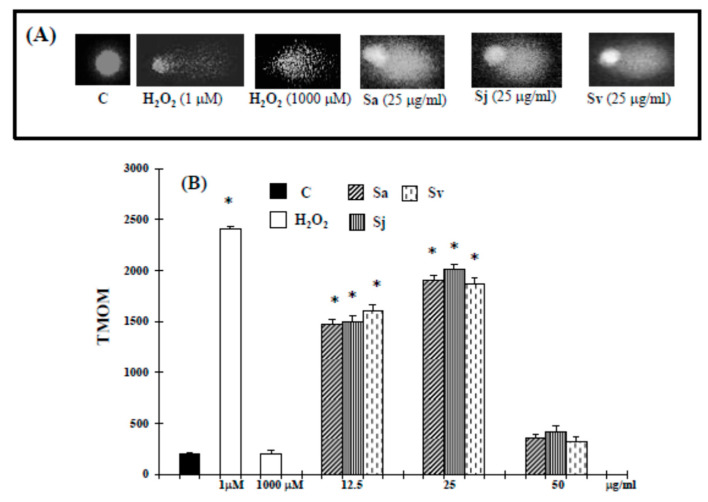
Comet assay of genomic DNA in DU-145 cancer cells untreated and treated with the essential oils from Sa, Sj and Sv for 72 h. Representative photomicrographs of microgel electrophoresed genomic DNA of untreated and treated cancer cells (**A**). TMOM values (**B**). TMOM = tail moment expressed as the product of TD (distance between head and tail) and TDNA (percentage of the fragmented DNA). The values are the mean ± standard deviation (SD) of three experiments performed in quadruplicate. * Significant vs. control untreated cells (*p* < 0.001).

**Figure 4:**
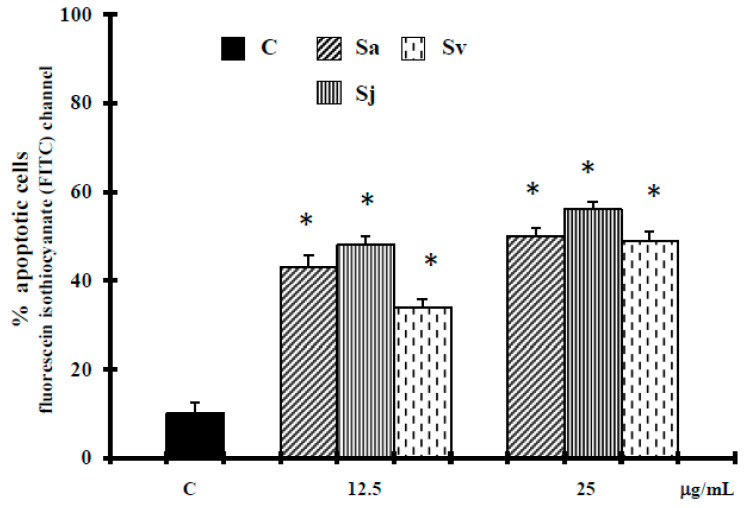
Terminal deoxynucleotidyl transferase (TdT)-mediated dUTP nick-end-labeling (TUNEL) assay in DU-145 cells untreated and treated with different concentrations of the essential oils from Sa, Sj and Sv for 72 h. The values are the mean ± standard deviation (SD) of three experiments performed in quadruplicate. * Significant vs. control untreated cells (*p* < 0.001).

Also the Tail moment (TMOM) values, as previously reported [29], suggest that the natural products at concentrations of 12.5–25 µg/mL trigger apoptotic cell death. Alternatively, in cells exposed to essential oils and H_2_O_2_ at higher concentrations, Comet assay did not evidence typical comet-like structures that occur during apoptosis (Figure 3). Terminal deoxynucleotidyl transferase (TdT)-mediated dUTP nick-end-labeling (TUNEL) results confirm the apoptotic process. In fact, the treatment of cells with essential oils at concentration of 12.5–25 µg/mL (Figure 4) induced a significant increase in green fluorescence, which is related to DNA fragmentation.

**Figure 5:**
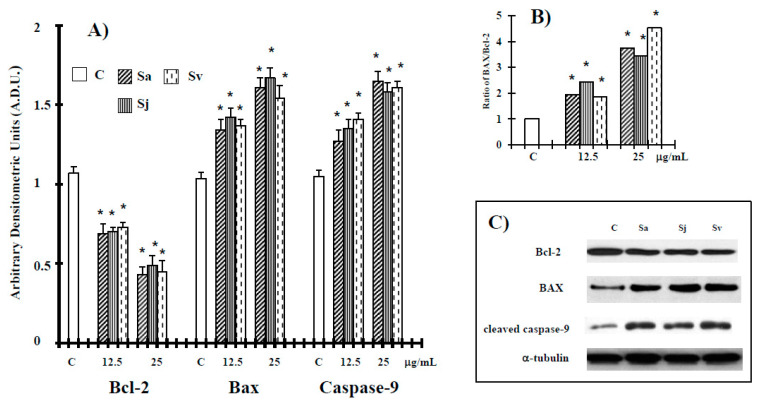
Levels of B-cell lymphoma 2 (Bcl-2), Bcl-2 associated X (Bax) and cleaved caspase-9 proteins in DU-145 cells untreated and treated with different concentrations of the essential oils from Sa, Sj and Sv for 72 h (**A**), and Bax/Bcl2 ratio (**B**). Representative blots of control and essential oils (25 µg/mL) are reported (**C**). The values are the mean ± standard deviation (SD) of three experiments performed in quadruplicate. * Significant vs. control untreated cells (*p* < 0.001).

During the intrinsic apoptosis process, apoptosis-related proteins such as Bcl-2 associated X (Bax), B-cell lymphoma 2 (Bcl-2), caspase-9 and caspase-3 are modulated for programmed cell death [30,31,32]. Therefore, we conducted Western blotting in DU-145 cells treated with the natural products for caspase-9, Bcl-2 and Bax. Natural products at 12.5 and 25 µg/mL concentrations suppressed anti-apoptotic protein Bcl-2 and increased cleaved caspase-9. The expression of Bax in DU-145 cell line has been debatable [33]. Reports indicate the expression of this protein in DU-145 cells [34,35], but other literature seems to indicate its absence [36]. In the present work, according to our previous studies in this cancer cell [37,38], also pro-apoptotic protein Bax was activated by the treatment with the three essential oils in DU-145 cells (Figure 5), shifting the Bax/Bcl-2 ratio in favor of apoptosis (Figure 5).

**Figure 6:**
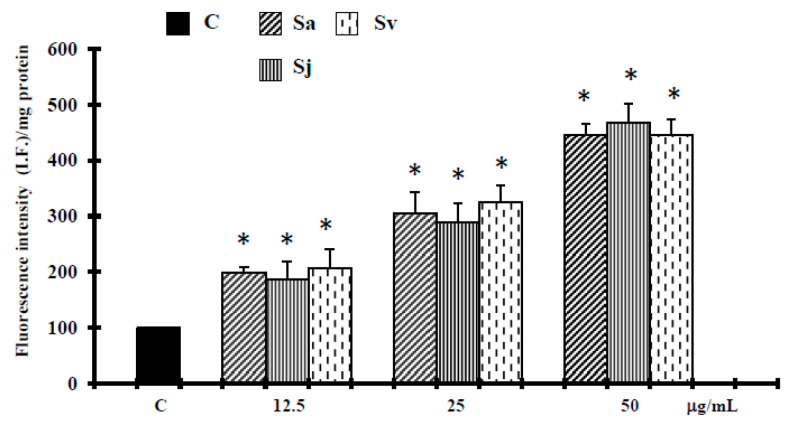
Reactive oxygen species (ROS) determination, performed by using a fluorescent probe 2′,7′-dichlorofluorescein diacetate (DCFH-DA), in DU-145 cells untreated and treated with different concentrations of the essential oils from Sa, Sj and Sv for 72 h. The values are the mean ± standard deviation (SD) of three experiments performed in quadruplicate. *Significant vs. control untreated cells (*p* < 0.001).

Reactive oxygen species (ROS) have been reported to be involved in cell death induced by a variety of stimuli [39,40], therefore the involvement of ROS production in the cell death induced by essential oils was examined. We found that the fluorescence of 2′,7′-dichlorofluorescein (DCF), the oxidized product of 2′,7′-dichlorofluorescein diacetate (DCFH-DA), increased significantly and in a concentration-dependent manner in the human cancer cells exposed to all essential oils (Figure 6).

**Figure 7:**
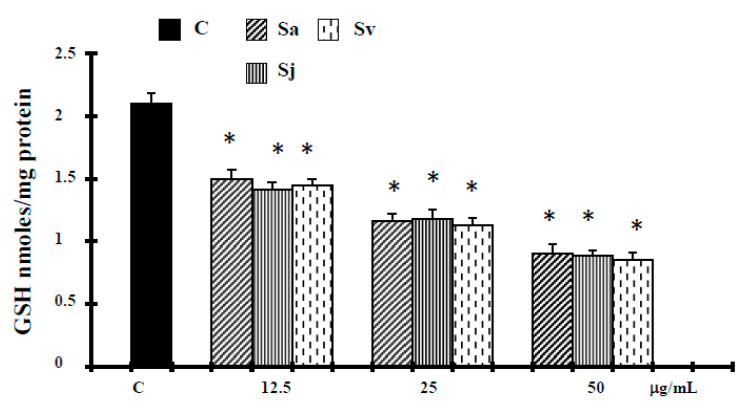
Level of Glutathione (GSH) in DU-145 cells untreated and treated with different concentrations of the essential oils from Sa, Sj and Sv for 72 h. The values are the mean ± standard deviation (SD) of three experiments performed in quadruplicate. *Significant vs. control untreated cells (*p* < 0.001).

Glutathione (GSH) acts as a reducing agent and as a major antioxidant within cells by maintaining a tight control of the redox status, therefore to evaluate the status of endogenous redox markers, glutathione levels were measured. In agreement with ROS levels, DU-145 cells showed a significant depletion of GSH content after the treatments with the natural products (Figure 7).

## 3. Discussion

Apoptosis is an essential cell process in homeostasis of multicellular organisms and its regulation has been involved in many human tumors, including prostate cancer [41]. Recently, it has been reported that caryophyllene oxide selectively affected cancer cells [42] and synergistically potentiated the paclitaxel anticancer activities in DU-145 human prostate cancer cell line [43]. Anticancer activities of caryophyllene oxide may be exerted through suppression of cellular growth and induction of apoptosis [44]. In particular, Park et al. [45] showed that this oxygenated sesquiterpene suppressed PC-3 prostate cancer cell proliferation in a dose-dependent manner. Moreover, it induced reactive oxygen species generation, Mitogen-activated protein kinase (MAPK) activation, and inhibition of PI3K/AKT/mTOR/S6K1 signaling pathway in these cells, a pathway which is essential in cell survival, proliferation, and angiogenesis of the tumor [45]. Furthermore, the same authors found that it significantly reduced levels of pro-cancer proteins, those involved in proliferation cyclin D1, metastasis COX-2 (cyclooxygenase 2), angiogenesis VEGF (vascular endothelial growth factor), and apoptosis inhibitors bcl-2, bcl-xL, IAP-1, IAP-2 (inhibitor of apoptosis 1 and 2), and surviving [46].

Here we report the biological activities against DU-145 cell line of Sa, Sj and Sv essential oils, all rich in oxygenated sesquiterpenes, with caryophyllene oxide as main compound (12.5%, 12.8% and 12.7% of total oils, respectively [22]). Interesting, according to our previous experimental evidence in melanoma cells, at non-toxic concentration in normal cells [22], all essential oils were able to inhibit the growth of prostate cancer cells (Figure 1), activating an apoptotic process at lower concentrations. In fact, an increase of caspase-3 enzyme activity occurred in DU-145 cells treated with all natural products at 12.5 and 25 µg/mL concentrations (Figure 2). The hypothesis of apoptosis induction in our experimental conditions was reinforced by a high DNA fragmentation (Comet assay) (Figure 3) and an increase in the percentage of apoptotic cells (TUNEL assay) (Figure 4), not correlated to LDH release (Table 1), a marker of membrane breakdown. Alternatively, we found a significant LDH release at higher concentration (50 µg/mL) (Table 1), suggesting that in these experimental conditions the three essential oils start a necrotic pathway in prostate cancer cells.

It has been demonstrated that the Bcl-2 family members regulate the intrinsic (mitochondrial mediated) apoptotic pathway; particularly, pro-apoptotic Bax and anti-apoptotic Bcl-2 are important for the cytochrome C release and the subsequent downstream activation of caspase protein [47]. Therefore, in the present work, the molecular mechanisms involved to such effects were further examined by evaluating the ability of the three essential oils to affect the expression level of cleaved caspase-9 and the mitochondrial-associated apoptotic proteins, Bcl-2 and Bax. The expression of Bcl-2 was decreased after the treatment with the essential oils at 12.5 and 25 µg/mL (Figure 5). By contrast, an increase in Bax protein, shifted the Bax/Bcl-2 in favor of apoptosis (Figure 5). Meanwhile, caspase-9 was shown to be observably activated (Figure 5). These results suggest that the cellular apoptosis elicited by all essential oils was related to the activation of the mitochondrial-associated pathway in DU-145 cells.

It has been suggested that the increase of ROS production, correlated to the release of cytochrome c from mitochondria or the depletion of endogenous antioxidants, can induce cell death for apoptosis [39,48]. According this hypothesis, the ROS production increase, at 12.5–25 µg/mL concentrations, associated with a decrease in glutathione levels (Figure 7), could amplify the apoptosis cascades. On the other hand, it has been reported that caryophyllene oxide increases reactive oxygen species from mitochondria, which in turn induce programmed cell death by the intrinsic apoptotic pathway [45]. Alternatively, at higher concentrations, when the antioxidant capacity of the cells is further reduced (Figure 7), our results seem to indicate that necrosis (Table 1) was induced by a more increase in these active species (Figure 6), generating intolerable oxidative stress in cancer cells that are already near a threshold for tolerating ROS [40].

Under these experimental conditions, the potential anticancer activity of each compounds present in the essential oils was not evaluated against DU-145 cell line, therefore at this stage it is not possible to say which of these compounds are responsible for the observed effects. However, on the base of literature data, it is possible to hypothesize that the biological effects exhibited by the essential oils from Sa, Sj and Sv could be related to cariophyllene oxide, found in comparable concentration in all samples, 12.5%, 12.8% and 12.7% of total oils, respectively [22]. However, as previously suggested [13,22,23], it is possible that the activity of the main components is also modulated by other minor molecules which may act synergistically. Carvacrol, one of the components of the essential oils from Sa, Sj and Sv, induces apoptosis in different cancer cell lines [22,49]. It has to be pointed out that thymol, occurring in the essential oils of Sj and Sv, is able to trigger programmed cell death by the intrinsic apoptotic pathway [50]. α-Humulene was found active against the prostate cancer cells LNCaP [51].

## 4. Materials and Methods

### 4.1. Essential Oils

Aerial parts from Sa, Sj and Sv were collected by N. A. Arnold at the full flowering stage from plants wild growing at El Kfour, Lebanon, in August 2012. Typical specimens (Sa/B, Sj/B and Sv/B respectively), leg. and det. N. Arnold s. n., confirm. Th. Raus, were deposited in the Herbarium of the Botanischer Garten, Berlin University. The air-dried samples were ground in a Waring blender and then subjected to hydrodistillation for 3 h using *n*-hexane as a solvent according to the standard procedure described in European Pharmacopoeia (2008) and as previously published [22]. The samples yielded (*w*/*w*) 0.28% of oil for Sa, 0.25% of oil for Sj and 0.30% of oil for Sv. Gas chromatography analysis of the most constituents has been published previously [22].

### 4.2. Study on Human Tumor Cell Line

#### 4.2.1. Cell Culture and Treatments

Human prostate cancer androgen-non responsive DU-145 cells were purchased from the American Type Culture Collection. DU-145 cells were maintained in Earle Minimal Essential Medium (EMEM), containing 10% fetal calf serum, 1 mM l-glutamine, antibiotics (50 IU/mL penicillin and 50 µg/mL streptomycin) and 1% non-essential aminoacids. The cells were plated at a constant density to obtain identical experimental conditions in the different tests, thus to achieve a high accuracy of the measurements. In the MTT assay, the cells were plated at 6 × 10^3^ cells per well for DU-145 cancer cells in a 96-well flat-bottomed 200 µL microplate. For other tests, the cancer cells were plated at 8 × 10^5^ cells (2 mL) per 35 mm culture dish. After 24 h incubation at 37 °C under a humidified 5% carbon dioxide to allow cell attachment, the cells were treated with different concentrations of the essential oils from Sa, Sj and Sv, no toxic for normal cells [22], and incubated for 72 h under the same conditions. Stock solution of oils was prepared in dimethyl sulfoxide (DMSO) and the final concentration of this solvent was kept constant at 0.25%. Control cultures received DMSO alone.

#### 4.2.2. MTT Bioassay

MTT assay was performed as described previously [22].

#### 4.2.3. Lactate Dehydrogenase (LDH) Release

LDH activity was spectrophotometrically measured in the culture medium and in the cellular lysates at 340 nm by analyzing nicotinamide adenine dinucleotide (NADH) reduction during the pyruvate-lactate transformation, as previously reported [22]. The percentage of LDH released was calculated as percentage of the total amount, considered as the sum of the enzymatic activity present in the cellular lysate and that in the culture medium.

#### 4.2.4. Activity of Caspase-3

The activity of caspase-3 was determined by using the Caspase colorimetric assay Kit (SIGMA RBI, St. Louis, MO, USA), as previously described [22]. The total protein content, used to reflect cell number and measured according to Bradford [52], was evaluated for each sample, and the results are reported as OD 405 nm/mg protein and compared to relative control.

#### 4.2.5. DNA Analysis by COMET Assay

The presence of DNA fragmentation was examined by single cell gel electrophoresis (COMET assay), according to Singh et al. [53], and as previously reported [22]. Hydrogen peroxide (H_2_O_2_) (30% *w*/*w*, Sigma Aldrich Co., St. Louis, MO, USA), an apoptotic inductor in cancer cell lines, was used as standard.

#### 4.2.6. TUNEL Assay (ApoAlert^®^ DNA Fragmentation Assay)

The nuclear DNA fragmentation was evaluated by a commercial kit (ApoAlert^®^ DNA fragmentation Assay, Clontech Laboratories, Inc., Mountain View, CA, USA) in accordance with the manufacturer’s instructions, as previously reported [54].

#### 4.2.7. Western Blot Analysis

The expression of Bcl-2, Bax and cleaved caspase-9 proteins was evaluated by western blot analysis, as previously described [48]. Bcl-2 (SAB2500154, Sigma Aldrich) (1:500 dilution), -Bax (B3428, Sigma Aldrich) (1:2000 dilution), -cleaved caspase-9 (AB3629, Sigma Aldrich) (1:500 dilution), and α-tubulin (T5326; Sigma Aldrich) (1:5000 dilution) antibodies were diluted in Tris Buffered Saline, 0.1% Tween 20 (TBST)and membranes incubated for 2 h at room temperature. Antibodies were detected with horseradish peroxidase-conjugated secondary antibody using the enhanced chemiluminescence detection Supersignal West Pico Chemiluminescent Substrate (Pierce Chemical Co., Rockford, IL, USA). Bands were measured densitometrically by ImageJ software (NIH, Bethesda, MD; available at http://rsb.info.nih.gov/ij/) and their relative density calculated based on the density of the α-tubulin bands in each sample. Values were expressed as arbitrary densitometric units corresponding to signal intensity.

#### 4.2.8. Reactive Oxygen Species Assay

Reactive oxygen species (ROS) determination was performed by using a fluorescent probe 2′,7′-dichlorofluorescein diacetate (DCFH-DA), as previously described [54]. The total protein content, measured according to Bradford [52] was evaluated for each sample, and the results are reported as fluorescence intensity/mg protein and compared to relative control.

#### 4.2.9. Measurement of GSH Levels

Glutathione (GSH) levels were measured as described previously [55]. Measurement of GSH by this method involves the oxidation of GSH by the sulfhydryl reagent 5,5′-dithio-bis(2-nitrobenzoic acid) (DTNB) to form the yellow derivative 5′-thio-2-nitrobenzoic acid (TNB), measurable at 412 nm.

#### 4.2.10. Statistical Analysis

Representative data from three independent experiments, performed in quadruplicate, are shown and quantitated, and represented as mean ± standard deviation (SD). Results were analyzed using one-way ANOVA followed by Dunnett’s post-hoc test for multiple comparisons with control. All statistical analyses were performed using the statistical software package SYSTAT, version 9 (Systat Inc., Evanston, IL, USA).

## 5. Conclusions

In conclusion, the present paper demonstrates that Sa, Sj and Sv essential oils, for their active components and in particular cariophyllene oxide, are able to reduce the growth of prostate cancer cells, activating an apoptotic process, correlated, at least in part, to modulation of redox-sensitive mechanisms. Therefore, this evidence suggests that these natural products can be considered potential candidates to be tested also in in vivo models, alone or in combination with chemotherapy agents, to provide a scientific support for the anticancer employ of Sa, Sj and Sv species in traditional herbal preparations and to hypothesize a possible use, in association with chemotherapy, for the management of prostate cancer. 

## Figures and Tables

**Table 1:** Lactate dehydrogenase (LDH) release in DU-145 cells untreated and treated with the essential oils from Sa, Sj and Sv at different concentrations for 72 h.

Treatments	DU-145 % LDH Released
Vehicle DMSO (control)	3.05 ± 0.7
H_2_O_2_	
1 µM	2.9 ± 0.7
1000 µM	49 ± 0.4 *
Sa	
12.5 µg/mL	4.23 ± 0.8
25 µg/mL	4.97 ± 0.6
50 µg/mL	16.4 ± 0.9 *
Sj	
12.5 µg/mL	4.95 ± 0.8
25 µg/mL	4.91 ± 0.6
50 µg/mL	16.3 ± 0.6 *
Sv	
12.5 µg/mL	4.03 ± 0.4
25 µg/mL	5.1 ± 0.7
50 µg/mL	17.1 ± 0.8 *

The values are the mean ± standard deviation (SD) of three experiments performed in quadruplicate. * The values were significant vs. control untreated cells (*p* < 0.001).

## Abbreviations

EMEM	Earle minimal essential medium
PBS	Phosphate buffer saline
Bcl-2	B-cell lymphoma 2
Bax	Bcl-2 associated X protein
MTT	3(4,5-dimethyl-thiazol-2-yl)2,5-diphenyl-tetrazolium bromide
LDH	Lactate dehydrogenase
Ac-DEVD-pNA	Acetyl-Asp-Glu-Val-Asp p-nitroanilide
HEPES	1-piperazineethane sulfonic acid, 4-(2-hydroxyethyl)-monosodium salt
CHAPS	3[(3-cholamidopropyl)dimethylammonio]-propanesulfonic acid
DTT	1,4 dithio-dl-threitol
TUNEL	Terminal deoxynucleotidyl transferase (TdT)-mediated dUTP nick-end-labeling
DCFH-DA	2′,7′-dichlorofluorescein diacetate
DCF	2′,7′-dichlorofluorescein
GSH	Glutathione
DTNB	5,5′-dithio-bis(2-nitrobenzoic acid)
TNB	5′-thio-2-nitrobenzoic acid
Sa	*Salvia aurea*
Sj	*Salvia judaica*
Sv	*Salvia viscose*
