# Correction: Russo et al. Involvement of Bax and Bcl-2 in Induction of Apoptosis by Essential Oils of Three Lebanese *Salvia* Species in Human Prostate Cancer Cells. *Int. J. Mol. Sci.* 2018, *19*, 292

**DOI:** 10.3390/ijms25168785

**Published:** 2024-08-13

**Authors:** Alessandra Russo, Venera Cardile, Adriana C. E. Graziano, Rosanna Avola, Maurizio Bruno, Daniela Rigano

**Affiliations:** 1 Department of Drug Sciences, University of Catania, V.le A. Doria 6, 95125 Catania, Italy; 2 Department of Biomedical and Biotechnological Sciences, Section of Physiology, University of Catania, Via S. Sofia, 89, 95123 Catania, Italy; cardile@unict.it (V.C.); acegraz@unict.it (A.C.E.G.); rosanna.avola@unict.it (R.A.); 3 Department of Biological, Chemical and Pharmaceutical Sciences and Technologies (STEBICEF), University of Palermo, V.le delle Scienze, Parco d’Orleans II, 90128 Palermo, Italy; 4 Department of Pharmacy, University of Naples Federico II, Via D. Montesano, 49, 80131 Naples, Italy; drigano@unina.it

In the original publication [1], there was a mistake in the preparation of Figure 3. In the COMET assay, the different photomicrographs of the microgel-electrophoresed genomic DNA of cells are very similar. An incorrect copy and paste had been performed. The corrected Figure 3 appears below. The authors state that the scientific conclusions are unaffected. This correction was approved by the Academic Editor. The original publication has also been updated.

**Figure 3:**
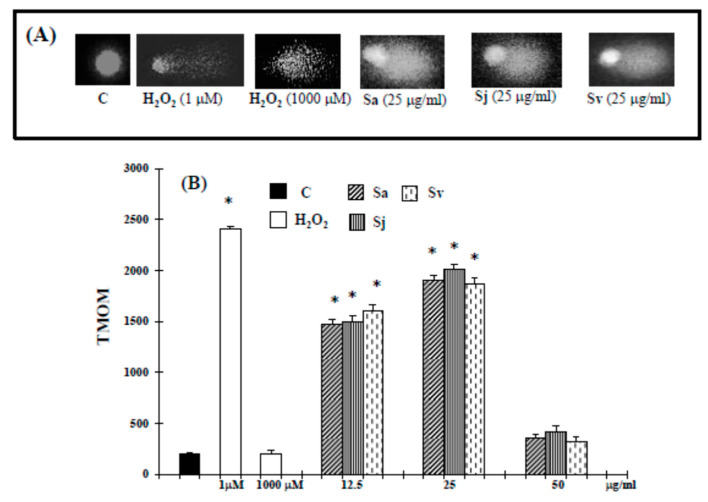
Comet assay of genomic DNA in DU-145 cancer cells untreated and treated with the essential oils from Sa, Sj and Sv for 72 h. Representative photomicrographs of microgel electrophoresed genomic DNA of untreated and treated cancer cells (**A**). TMOM values (**B**). TMOM = tail moment expressed as the product of TD (distance between head and tail) and TDNA (percentage of the fragmented DNA). The values are the mean ± standard deviation (SD) of three experiments performed in quadruplicate. * Significant vs. control untreated cells (*p* < 0.001).
